# Construction Notes

**Published:** 1893-12-30

**Authors:** 


### CONSTRUCTION NOTES.

### Chertsey New Infectious Diseases Hospital.

The site for this building, which was erected by the Chertsey Union Rural Sanitary Authority, is at Ottershaw, a short distance from the workhouse. Facing the entrance gate is the administrative block, which consists of the medical officer's and visitors' rooms, matron's parlour, kitchen, stores, scullery, larder, and various offices on the ground floor. Above is the matron's and nurses' bed-rooms, and servants' ditto. The isolation block, which is reserved for the reception of typhoid and diphtheritic cases, is at the rear, and to the right of the building just described. It consists of a one-storey building, 96 feet long by 30 feet wide. The diphtheria ward for females is lofty and airy. Indeed, the latter remark applies to all wards in the hospital. The wards occupied by the two sexes are divided by a spacious hall, in which there is a bath-room. In the kitchen is one of Herring's well-known kitcheners. From the kitchen the nurses, when engaged in culinary operations, are able to command a view of every bed in the wards through a small but ingeniously-constructed window. The typhoid wards are designed to accommodate two adults, the sexes again being divided by an entrance hall, bath-room, and kitchen. The whole of the wards in the isolation block are heated by Herring's register stoves. Facing the isolation wards is the scarlet fever block, the female ward being airy and lofty. It contains beds for eight adult patients. The male ward contains four beds for adults; male and female wards are divided in a similar manner to those in the isolation block, and the arrangements are just as complete in every respect. A building containing washhouse and disinfecting room is situated some distance from the scarlet fever wards; the disinfecting house being fitted with one of Washington Lyons' patent steam disinfectors, and the washing-house by a Bradford washing machine and wringer. The mortuary, ambulance shed, and workshop are included in a small building near the entrance gate, provision having been made for a thick belt of trees to be placed near. The whole of the buildings will soon be invisible from the road. The architectural designs were furnished by Mr. C. Welsh, of Chertsey. The contractors were Messrs. Knight and Sons, whose tender amounted to £3,900.

### New Small-pox Hospital for Leeds.

When built, this hospital, in details and arrangements, will be like the one at Newcastle, which commended itself most to the Building Committee. It will be on the pavilion system, one storey high, with a ward at one end for males and at the other end for females, the two being separated by the nurses' department, &c. The building will comprise 30 beds for patients, and 12 for those connected with the administrative department. The plans were designed by Mr. Thomas Hewson, City Engineer of Leeds.

### New Hospital at Brighton.

Plans for the construction of a new hospital, to supplement the existing Brighton, Hove, and Sussex Throat and Ear Hospital, have been accepted from Messrs. Scott and Cawthorne, of Regency Square. The design provides for a three-storied house, with ground floor and basement. On the ground floor there will be a large consulting-room, 21 feet by 23, and a hall running the whole length of the building. This hall will be used as a waiting-room until the last part of the building is completed. It is arranged that two-thirds of the hospital will be built first, leaving the other third till the state of funds permits. The completed portion will, however, make a very workable hospital. On the first floor the female ward will be situated. It will be 31 feet by 25, and will hold nine beds. The operation-room is large and lofty, and there will be an etherising chamber, a nurses' parlour and bed-rooms. The male ward will be on the second floor, with two isolating wards and a nurse's bed-room. The house surgeon's room will also be on this floor. The third floor will provide a kitchen, servants' bed-rooms, a scullery, and other rooms, the basement supplying coal-cellars, lavatories, &c.

### New Small-pox Hospital at Birmingham.

The plans for this hospital were prepared by Mr. W. H. Ward, the architect of the Workhouse Infirmary. This is designed on the pavilion style, and will accommodate about 240 patients. The buildings are estimated to cost £41,000. Provision is made for ten pavilions, with an administrative building, and such part of the nurses' home and servants' quarters as was necessary to secure the requisite accommodation. The increasing number of patients are now treated under disadvantageous circumstances, and as soon as the pavilions can be opened they will be put to immediate use. Each building is 200 feet long, and contains two wards, which are separated by the entrance hall and waiting-room. The baths, lavatories, and kitchens are situated at each end of the pavilion, and the utmost care has been taken with arrangements to secure perfect isolation. Each pavilion will have its own system of drainage, and will be further supplied independently with water. That part of the scheme which is now being completed is at the corner farthest from the road, and the administration for a time will have to be conducted from within the pavilion instead of from a separate building.

